# Renewable fuel gases and important organic compounds production from ethanol dehydrogenation using nickel oxide, a green-synthesized catalyst

**DOI:** 10.1007/s11356-026-37421-8

**Published:** 2026-04-16

**Authors:** Carla Maria Beraldi Gomes, Denise Aparecida Zempulski, Caroline da Ros Montes D’Oca, Helton José Alves

**Affiliations:** 1 Renewable Materials and Energy Laboratory (LABMATER), Federal University of Paraná, UFPR - Setor Palotina, Rua Pioneiro, 2153, Dallas, Palotina, PR Brazil; 2 Medicinal and Agrochemical Organic Synthesis Research Group (SOMA), Federal University of Paraná, Rua Col. Francisco H. Dos Santos, 100, Jardim das Américas, Curitiba, PR Brazil

**Keywords:** Ethanol dehydrogenation, Fuel gases, NiO nanoparticles, Organic compounds, Catalyst crystal structures

## Abstract

Bioethanol dehydrogenation is a promising route for clean and renewable hydrogen production, aligning with low-carbon economy goals. Additionally, it promotes the synthesis of valuable organic compounds from bioethanol. This study investigated the use of green-synthesized NiO nanoparticles as a catalyst for ethanol dehydrogenation. The highest selectivity for hydrogen production (97.4%) was achieved using 10% NiO at 260 °C. However, at 10% NiO and 280 °C, the fuel gas mixture yielded a higher calculated calorific value (1,231.6 kJ). This condition was used to evaluate the reaction’s kinetic over a period of 1 to 8 reaction-hours. During these experiments, a progressive increase in gas volume was observed, along with a transition in the gas products from propane to hydrogen, followed by methane. These changes correlated with ethanol conversion to organic compounds (1,1-diethoxyethane and 1-butanol) in the liquid phase, as well as catalyst’s crystal structure alterations, from Ni^2+^ face-centered-cubic (FCC) to metallic nickel FCC and hexagonal close-packed (HCP) phase.

**Supplementary Information:**

The online version contains supplementary material available at 10.1007/s11356-026-37421-8.

## Introduction

In the global energy scenario, the conversion of biomass from abundant and renewable resources to clean energy with low carbon emissions is an important and urgent demand, representing one of the major challenges in scientific research (Bach et al. 2022; Chatterjee et al. 2021). Bioethanol is a consistent, sustainable, and clean biofuel that is commonly used as an additive to gasoline in many countries, such as Brazil, the United States, and China. It can be produced by the fermentation process of glucose and starch (Pang et al. 2016; Sun and Wang 2014). In this context, upgrading bioethanol to more valuable chemicals are of interest, given that it is derived from a natural source with a large supply-chain.

Hydrogen (H_2_) is recognized as an environmentally friendly and sustainable alternative, a renewable energy carrier that aligns with low carbon economy policies (Chatterjee et al. 2021; Huang et al. 2021). It is primarily produced through biomass gasification, methane reforming, and catalytic ethanol steam reforming, where the main gas-phase products are H_2_ and CO (synthesis gas). However, these processes require high energy consumption due to purification measures and high-temperature reactions (Dal Santo et al. 2012). Therefore, the production of renewable H_2_ from catalytic bioethanol dehydrogenation has attracted interest as a potential low-cost and more sustainable process (Chatterjee et al. 2021; Wismann et al. 2019).

One of the key advantages of catalytic ethanol dehydrogenation is the relatively low reaction temperature, which occurs between 250 and 300 °C, which is much lower than the temperature required for ethanol steam reforming (550–700 °C with catalysts) under atmospheric pressure. The lower temperature in catalytic ethanol dehydrogenation is advantageous in terms of reduced energy consumption and a diminished risk of catalyst deactivation due to coking and/or sintering (Bach et al. 2022; Bshish et al. 2011).

The ethanol dehydrogenation process is also an economical and environmentally friendly alternative to other conventional commercial processes. For example, the synthesis of acetaldehyde and 1-butanol, which are important starting materials for the synthesis of various industrial chemicals (Ob-eye et al. 2019; Sun and Wang 2014; Wu et al. 2018). Commonly used methods for acetaldehyde production involve routes such as partial oxidation of ethane, hydration of acetylene, oxidation of ethylene, and oxidative dehydrogenation of ethanol (Ob-eye et al. 2019). 1-Butanol, a valuable additive for gasoline due to its properties—such as higher energy density (~ 90% of that of gasoline), lower water adsorption, higher air-to-fuel ratio, and lower heat of vaporization—is traditionally manufactured from fossil-based materials using oxo process by applying homogeneous cobalt and rhodium catalysts (Wu et al. 2018). A promising and more sustainable synthesis route for 1-butanol is from ethanol, through the Guerbet reaction, which involves the use of acetaldehyde as starting material, straightforwardly produced from ethanol dehydrogenation (Lopez-Olmos et al. 2020).

In this context, the development of catalysts is fundamental to the ethanol dehydrogenation process for hydrogen and acetaldehyde production. Some nickel catalysts have been successfully employed, and among the advantages of their uses are considerable cheap, harmless, and stable metals when replacing noble metals, as presented in Table 1 (Shan et al. 2017).

**Table 1:** Nickel catalysts applied to ethanol dehydrogenation process

Catalyst	Reaction Conditions	Products	Authors
NiO@SBA-15	300–400 °C, atmospheric pressure	H_2_, CO, CO_2_, CH_4_, ethane, ethylene, propane, propylene, butene, ethylene, diethyl ether, benzene	Chatterjee et al. 2021
Ni-MgAlO	200–275 °C	H_2_, CO, CO_2_, CH_4,_ acetaldehyde, butanal, *n*-butanol, hexanol, octanol, diethyl ether, ethylene, 1,1-diethoxy ethane, 2-ethyl butanol, ethyl butyrate	Pang et al. 2016
Ni/ACC (Activated Carbon Catalyst)	250—400 °C, atmospheric pressure	Acetaldehyde and ethylene	Ob-eye et al. 2019
NiCu alloy	200—350 °C, atmospheric pressure	H_2_, CO, CO_2_, acetaldehyde	Shan et al. 2017

Focused on hydrogen production, in the study developed by Bach et al. (2022), NiO nanoparticles were synthesized and effectively utilized in the ethanol dehydrogenation process. The catalyst was obtained by a consistent green synthesis approach, employing a modified Pechini method with chitosan as the polymeric precursor (Mariani et al. 2017). Bach et al. (2022) evaluated the activity of the NiO catalyst in three concentration levels for the dehydrogenation of ethanol, performed at three distinct reaction temperatures. The gaseous composition and volume content were also investigated in a kinetic study at 260 °C over an eight-hour period, employing individual reaction tests. In general, it was observed an excellent selectivity for hydrogen as the major component in the gaseous phase, with a significant volume of gases produced. Furthermore, it was also observed a fascinating result regarding the catalyst, which suffered changes in its crystal structure during the reaction. Analysis of the liquid content using gas chromatography indicated possible structures formed by the dehydrogenation reaction of ethanol (Bach et al. 2022).

Based on the study developed by Bach et al. (2022) the objective of this research is to further explore ethanol dehydrogenation using the NiO nanoparticles catalyst under new reaction conditions. In this context, in addition to improving hydrogen production and other fuel gases, this study also seeks to gain a deeper understanding of the more valuable compounds produced in the liquid fraction and how it is related to the crystal structure change of the catalyst.

## Materials and methods

### General information

Commercially available standard materials were used without further purification. Ethanol (99.5%, Synth), ethyl acetate (HPLC, J. T. Baker); 1,1-diethoxyethane (99%, Sigma Aldrich), *n*-butanol (P.A., Êxodo), methylcyclopentane (97%, Sigma Aldrich), 2-pentanone (98%, Sigma Aldrich). The synthesis and characterization of NiO nanoparticles catalyst employed was previously described by Bach et al. (2022).

### Catalytic tests

An experimental design was elaborated based on the previous study developed by Bach et al. (2022) to investigate the effects of temperature and catalyst content on the dehydrogenation of ethanol. The experiments are summarized in Table 2, with three different levels of each variable, using 15 g de ethanol for 4 h of reaction. The volume and content of the gaseous phase, calorific power of the generated product, and components of the liquid fraction of each experiment were analyzed.

**Table 2:** Experimental conditions for dehydrogenation reactions of ethanol with NiO catalyst

Reaction code	Temperature	Catalyst Content
R1	240 ºC	6%
R2	240 ºC	10%
R3	240 ºC	14%
R4	260 ºC	6%
R5	260 ºC	10%
R6	260 ºC	14%
R7	280 ºC	6%
R8	280 ºC	10%
R9	280 ºC	14%

^*^All reactions were performed with 15 g ethanol for 4 h

Catalytic tests were performed following the experimental protocol from our previous study, using the conditions specified in Table 2. A kinetic study was also conducted, with reaction times ranging from 1 to 8 h, in individual tests. After each reaction, the reactor cooled to room temperature over 8 h. The gaseous, liquid, and solid fractions were collected for further analysis and characterization.

### Characterization analysis of the gaseous fractions

The gaseous phase was collected in glass ampoules, and the sample volume was determined by multiplying the ampoule volume by the number of ampoules used. Analysis of the gaseous product was carried out using a gas chromatography (C2V-200 micro-GC, Thermo Scientific) equipped with a nano TCD detector, an MS5A (molecular sieves 5 Å) PLOT column, and a U-Bond (divinylbenzene type U) column. Argon gas (> 99.999% purity) was utilized as the carrier gas during the analysis.

#### Calorific power calculations

Calorific power of each catalytic test (R1-R9) was calculated using the real gas law—van der Waals equation (Eq. 1), and the Higher Calorific Value (HCV) of the produced gases (The Engineering Toolbox 2005).

1 $$\left(P+\frac{{an}^{2}}{{V}^{2}}\right)\left(V-nb\right)=nRT$$

where *P* is the reactor pressure at room temperature, measured after the reaction was completed; *V* is the gas volume of the produced gas at room temperature, *n* is the number of mols of the produced gas; *R* is the universal gas constant and *T* is the temperature of the gas. The constants *a* and *b* are van der Waals constants, being *a* correction for the intermolecular attractive forces and *b* a correction for the effective volume occupied by gas particles. Each gas has individual values for *a* and *b* (The Engineering Toolbox 2005).

To calculate the calorific power of the mixture of gases it was applied the following Eq. 2.

2 $$Cp\left(total\right)=\left(Cp\left(gas a\right). m \left(gas a\right)\right)+\left(Cp\left(gas b\right). m\left(gasb\right)\right)+\dots$$

where, *Cp*(total) is the calorific power of the gas mixture; *Cp*(gas a), *Cp*(gas b), etc., are the HCV of the individual gases in the mixture, and* m*(gas a), *m*(gas b), etc., are the masses of each gas (The Engineering Toolbox 2005).

### Characterization analysis of the liquid fractions

The liquid phase, obtained for each reaction after centrifugation, was stored at a low temperature (−10 ºC), in a freezer. The liquid samples were analyzed by using gas chromatography (GC–MS and GC-FID) and spectroscopic (NMR) techniques. A Karl Fischer analysis was performed to determine the humidity content.

#### Gas Chromatography–Mass Spectrometry (GC-MS) analysis

GC–MS identification data of liquid fractions were performed on a Shimadzu QP-2010 SE gas chromatography coupled with a mass spectrometer detector with electron impact ionization (70 eV) using a DB-23 ms column (60 m × 0.25 mm DI. × 0.25 μm film, Agilent J&W Columns, Santa Clara, CA, USA). For the analysis were used the following conditions: split/splitless inlet with split ratio of 1:120; inlet temperature: 230 °C; pressure: Helium (He) at 89.5 kPa; septum purge: 3 mL min^−1^; sample size: 1 µL injection; initial column flow: 0.8 mL min^−1^ (constant flow with an average linear velocity of 22.8 cm sec^−1^); MS temperature transfer line: 230 °C; MS detector temperature: 230 °C; oven temperature program: starting at 40 °C for 15 min, followed by a ramp of 10 °C min^−1^ to 230 °C, and held for 10 min; MS program: start acquisition time 1: 2.50 to 6.80 min; acquisition time 2: 7.50 to 43.0 min; solvent cut: 2 min.

#### Gas Chromatography - Flame Ionization Detector (GC-FID) analysis

GC-FID quantification data of the components from liquid fractions were performed on a Shimadzu QP-2010 gas chromatography with a flame ionization detector using the same column used on GC–MS analysis, a DB-23 ms column (60 m × 0.25 mm DI. × 0.25 μm film, Agilent J&W Columns, Santa Clara, CA, USA). For the analysis were used the following conditions: split/splitless inlet with split ratio of 1:50; inlet temperature: 250 °C; pressure: He at 134.9 kPa; septum purge: 3 mL min^−1^; sample size: 1 µL injection; initial column flow: 1 mL min^−1^ (constant flow with an average linear velocity of 20.4 cm sec^−1^); oven temperature program: starting at 40 °C for 15 min, followed by a ramp of 10 °C min^−1^ to 100 °C, and held for 10 min; H_2_ flow: 40 mL min^−1^; air synthetic flow: 450 mL min^−1^; make-up flow: 30 mL min^−1^.

#### GC-FID Quantitative analysis

Working solutions of ethanol at concentrations of 3.43, 6.86, 10.28, 13,72, and 17.14 mmol mL^−1^ were prepared by diluting ethanol P.A. in ethyl acetate HPLC. A stock standard solution of 1,1-diethoxyethane at 2.58 mmol mL^−1^ was prepared in ethanol, and then five working solutions at concentrations of 0.001, 0.01, 0.10, 0.41, 0.72, and 1.03 mmol mL^−1^ were prepared from the stock solution. A stock standard solution of 1-butanol at 2.94 mmol mL^−1^ was prepared in ethanol, and then five working solutions at concentrations of 0.001, 0.01, 0.12, 0.47, and 1.17 mmol mL^−1^ were prepared from the stock solution. For all three standards, this procedure was performed in triplicate to construct the analytical curves. Without any prior treatment, 1 mL of the liquid fractions were collected on vials and then analyzed by GC-FID. The observed areas were used to calculate the concentrations of ethanol, 1,1-diethoxyethane, and 1-butanol, determining the percentage content of each component in the fraction.

#### Nuclear Magnetic Resonance (NMR)

^1^H and ^13^C{H} NMR spectra were recorded on a Bruker AVANCE 400 NMR spectrometer (Bruker, Karlsruhe, Germany), which was operated at 9.4 T (^1^H at 400 MHz and ^13^C at 100 MHz). NMR spectra of liquid fractions were acquired without an internal standard. The chemical shifts (δ) were reported in parts per million (ppm) relative to the ethanol signal. NMR spectra of the synthetic standards in CDCl_3_ had the chemical shifts (δ) reported in parts per million (ppm) relative to TMS as an internal standard. The spectra were interpreted as follows: s = singlet, br s = broad singlet, d = doublet, dd = doublet of doublet, t = triplet, td = triplet of doublet, q = quartet, m = multiplet. The coupling constants (*J*) are expressed in Hertz (Hz).

#### Karl Fischer analysis

The water content of selected samples was determined by volumetric Karl Fischer method (NBR11348-1) using a Volumetric Karl Fischer Titration V20 (Mettler—Toledo) (Associação Brasileira de Normas Técnicas 2018). The results were expressed in percentage by mass (%).

### Characterization analysis of the used NiO nanoparticle catalyst

The NiO catalysts utilized in the reactions were collected after the removal of the gaseous fractions. The separation of the liquid and solid phases was achieved through centrifugation. Subsequently, the catalyst was dried at 90 °C until a constant weight was obtained and then analyzed by XRD technique.

#### X-ray diffraction (XRD)

The samples were prepared as powder and analyzed in the range 5° < 2θ < 80° using Cu Kα radiation (λ = 1.54 Å, 45 kV, 25 mA), at a scanning rate of 1° min^−1^, on a Bruker D2 PHASER diffractometer. The crystallite diameter was determined using the Scherrer equation (Eq. 3):

3 $$D= \frac{K\lambda }{\beta \mathrm{cos}\left(\theta \right)}$$

where, *D* represents the mean crystallite diameter (Å), *K* is the Scherrer coefficient (0.9), *λ* is the X-ray wavelength (0.154 nm), *β* is the full width at half maximum (FWHM), and *θ* is the diffraction angle (Mark et al. 1985).

## Results and discussion

Reaction conditions for catalytic tests were established based on a previously developed study by our research group (Bach et al. 2022). The current study evaluated NiO nanoparticles catalyst on ethanol dehydrogenation at the conditions described in Table 2.

### Catalytic tests and analysis of the gaseous products

**Fig. 1:**
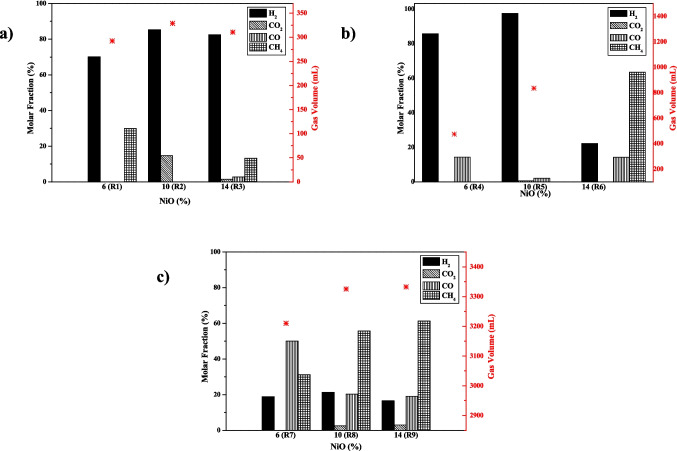
Chemical compositions and volumes of the gaseous products produced from the dehydrogenation reactions of ethanol, for 4 h, with diverse catalytic content, at (**a**) 240 °C, (**b**) 260 °C, and (**c)** 280 °C

Figure 1 presents the results from the gas chromatographic analysis of gaseous products generated during ethanol dehydrogenation.

For the reaction tests at 240 °C (Fig. 1(a)), an increase in hydrogen content was observed, with production rising from 70.1% in reaction R1 to 85.3% in R2, as the catalyst amount was raised from 6% (R1) to 10% (R2). However, further increase in the catalyst loading from 10% (R2) to 14% (R3) did not result in a significant change in hydrogen concentration, which remained at 82.5% in R3. These results indicated that, at 240 °C, it was not advantageous to increase the catalyst content from 10 to 14% since no considerable rise in hydrogen generation was observed.

Analyzing the results shown in Fig. 1(b) for the tests at 260 °C, a similar behavior was observed using 6% (R4) and 10% (R5) of the catalyst, as compared to R1 and R2 at 240 °C. Within the same temperature range, the first 4% increment in catalyst loading led to an increase in hydrogen content, from 85.7% (R4) to 97.4% (R5). However, in contrast to R2 and R3 at 240 °C, the second increment in catalyst content from 10% (R5) to 14% (R6) resulted in a change in the composition of the gaseous phase, with methane becoming the major gaseous component.

The last set of catalytic tests was carried out at 280 °C under the same conditions as previously evaluated (Table 1). Using 6% of NiO (R7), most of the gaseous fraction was composed of CO (50.0%) and CH_4_ (31.2%), with H_2_ as the minor component (18.8%) (Fig. 1(c)). However, in comparison with the previous conditions tested (R1-R6), the highest volume of gaseous products (3,210 mL), was achieved in R7. Increasing the catalyst loading from 6% (R7) to 10% (R8), H_2_ continued to be the minor component in the gaseous fraction, but the proportions of CO (20.3%) and CH_4_ (55.8%) changed, indicating that the increment of catalyst favored methane production over carbon monoxide. Further increasing the catalyst by 4%, from 10% (R8) to 14% (R9), did not significantly affect either the composition or the volume of the gas produced.

Based on the results presented in Fig. 1(a-c), higher temperatures resulted in larger gas volumes. Despite hydrogen being present in smaller proportion compared to other gases, R6 to R9 conditions facilitated an overall increase in gas formation, particularly in terms of volume. Analyzing the reactions with 10% NiO, at 260 °C (R5) and at 280 °C (R8), it was observed that R5 yielded the highest hydrogen content of 97.4% (813 of 835 mL). R8 test exhibited a hydrogen content of only 21.3%, but due to a larger total gas volume, this percentage corresponded to 710 of 3,325 mL of hydrogen. These results indicated that both reaction conditions produced a significant amount of hydrogen, but at a higher temperature, other gaseous components were also produced.

From Fig. 1,

the volume of each gas produced was determined using the molar fraction and the total volume. At the end of each experiment, after reaching the room temperature, the final pressure of the reactor was measured. The mass of each gas produced was then calculated by applying van der Waals equation (Eq. 1) (Table S1 – Supplementary Information). According to the literature, among the gases produced in the mixture, H_2_ and CH_4_ have HCV of 141.7 kJ/g and 55.5 kJ/g, respectively (The Engineering Toolbox 2005). Based on the calculated mass produced of those in each experiment, the total calorific was calculated using Eq. 2 for all conducted experiments (Table S1 – Supplementary Information). For experiments R1 – R6, the calorific power value obtained were relatively low, not exceeding 71.2 kJ. However, for experiments R7, R8, and R9, the calorific values were: 569.5, 1,231.6, 1,313.6 kJ, respectively. Although the molar fraction of H_2_ obtained at 280 ºC were lower than R1-R6, the gas volume was higher. Furthermore, the tests for R7-R9 produced gas with high calorific power, making them potential candidates for use as fuel sources.

The experimental plan (Table 2) was designed to promote ethanol dehydrogenation, which produces acetaldehyde and hydrogen, as shown in Eq. 4 (Huang et al. 2021). Based on the experiments conducted, it was observed that from R1 to R5, the expected favorability for ethanol dehydrogenation was observed, with H_2_ being the major component of the gaseous fraction. However, other gases such as CH_4_, CO_2,_ and CO were also observed under these conditions. The formation of CO is attributed to the dehydration of ethanol, which leads to the formation of ethylene (Eq. 5) (Zhang and Yu 2013), which in the presence of H_2_O, ethylene undergoes further decomposing to CO (Eq. 6) (Cazula et al. 2020). The water–gas shift (WGS) reaction yields CO_2_ and H_2_ (Eq. 7) (Ratnasamy and Wagner 2009). The formation of CH_4_ arises from the decomposition of acetaldehyde, as described in Eq. 8, which also furnishes CO (Ni et al. 2007).

4 $${\mathrm{C}}_{2}{\mathrm{H}}_{5}\mathrm{OH}\rightleftarrows {\mathrm{C}}_{2}{\mathrm{H}}_{4}\mathrm{O}+{\mathrm{H}}_{2}$$

5 $${\mathrm{C}}_{2}{\mathrm{H}}_{5}\mathrm{OH}\rightleftarrows {\mathrm{C}}_{2}{\mathrm{H}}_{4}+{\mathrm{H}}_{2}\mathrm{O}$$

6 $${\mathrm{C}}_{2}{\mathrm{H}}_{4}+{2\mathrm{H}}_{2}\mathrm{O}\to 2\mathrm{CO}+4{\mathrm{H}}_{2}$$

7 $$\mathrm{CO}+{\mathrm{H}}_{2}\mathrm{O}\rightleftarrows {\mathrm{CO}}_{2}+{\mathrm{H}}_{2}$$

8 $${{\mathrm{C}}_{2}{\mathrm{H}}_{4}\text{O }\to }_{ }{\mathrm{CH}}_{4}+\text{ CO}$$

Besides the ethanol utilized may contain a small quantity of water, during the dehydrogenation reaction, a reduction of Ni^2+^ to Ni^0^ (metallic) takes place, promoted by the presence of H_2_ atmosphere (Doppiu et al. 2004). Hydrogen acts as a reducing agent and leads to water elimination. This water eliminated can be used in the WGS process, which is responsible for CO_2_ production (Ni et al. 2007).

Even though H_2_ was not the predominant gas produced from R6 to R9, a large volume of total gas was observed in comparison to R1 to R5. The significant amount of H_2_ leads to in situ activation of the catalyst (Van der Borght et al. 2015), resulting in the production of water, which in combination with ethylene results in CO (Eq. 6) (Cazula et al. 2020). Under these conditions, the decomposition of acetaldehyde was also promoted, furnishing CH_4_ and CO (Eq. 8), the main gases observed at R6 to R9 tests (Doppiu et al. 2004).

**Fig. 2:**
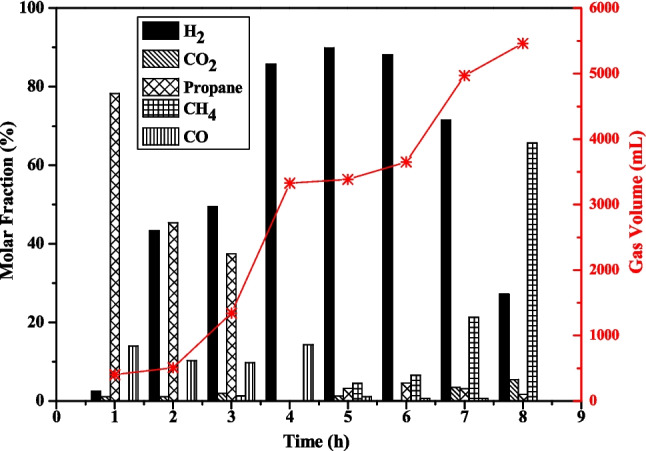
Chemical compositions and volumes of the gaseous products produced from the dehydrogenation reactions of ethanol at the kinetic study, with 10% of NiO catalyst at 280 °C

Based on the performed catalytic tests, R8 condition (10% NiO, at 280 °C) was selected for kinetic study, due to its high calculated calorific power associated with H_2_ molar fraction. Figure 2 presents the composition and volume of gases produced in eight individual experiments conducted to evaluate the reaction performance over reaction time.

The H_2_ formation starts from one hour of reaction, initially in low percentages, and then increases from two hours onwards. With one hour of reaction, 402.0 mL of gases were produced, which propane was the predominant gas with 78.3%, then CO (14.0%), H_2_ (2.5%), and a slight portion of CO_2_ (1.1%). In the second experiment with two hours of reaction, a significant increase in H_2_ content was observed (43.3%), close to propane (45.3%), CO_2_ (1.1%) and CO (10.3%), but no substantial growth in gases volume was observed, of 509.6 mL. Whitin a three-hour reaction time, H_2_ became the major component of gaseous fraction, and a high increase in gas volume was achieved, with 1,339 mL. The content of CO_2_ (1.9%) and CO (9.8%) remained consistent, while CH_4_ was introduced at a concentration of 1.3%.

In the study developed by Bach et al. (2022), propane was not detected on the conducted experiments. However, the formation of propane from ethanol had already been described in the literature. For example, Chatterjee et al. (2021) studied the conversion of ethanol to hydrogen and hydrocarbon fuels such as methane and butene over NiO@SBA-15 catalyst at a temperature range of 300 °C to 400 °C. The mechanism involved in ethanol-to-hydrocarbon conversion is considerably complex and it is based on ethanol-to-hydrocarbon (ETH) reactions, which involve the hydrocarbon pool (HCP) mechanism—a continuous rearrangement, addition, and cracking of HCP molecules. Van der Borght et al. (2015) proposed a ETH reaction mechanism over modified ZSM-5 by ethylene oligomerization to produce larger hydrocarbon molecules, which can subsequently undergo cracking or hydrogen transfer to give aromatics and paraffin compounds.

**Fig. 3:**
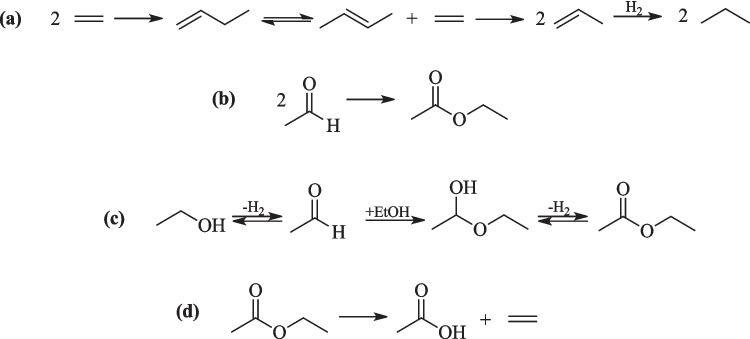
Reaction pathways of propane synthesis (**a**) ethylene formation by dehydration of ethanol and subsequent reaction steps, (**b**) ethyl acetate formation by Tishchenko reaction, (**c**) ethyl acetate formation by nucleophilic addition of ethanol, (**d**) decomposition of ethyl acetate furnishes ethylene to propane mechanism

If the ethanol dehydration (Eq. 5) mechanism occurs at the beginning of the kinetic experiment, the resulting ethylene molecule could potentially follow the HCP mechanism, as proposed by Iwamoto et al. (2011) in their study on ethanol conversion in polyolefins using a NiO/MCM-41 catalyst. It proposed that dehydration and isomerization mechanisms took place on the acidic silica support, while dimerization and metathesis on Ni site (Iwamoto et al. 2011). Based on that, one of the propane synthesis pathways considers ethylene formation. The proposed mechanism described in the literature, catalyzed by acid/basic sites of NiO catalyst, with hydrogenation of propene by H_2_ atmosphere (Fig. 3 (a)).

Another mechanism for ethylene formation involves ethyl acetate, which is formed from acetaldehyde—one of the products from ethanol dehydrogenation. Ethyl acetate can be generated throughout Tishchenko reaction (Fig. 3(b)) or by the nucleophilic addition of ethanol to acetaldehyde, followed by the dehydrogenation of the resulting hemiacetal 1-ethoxyethanol (Fig. 3(c)). The decomposition of ethyl acetate into ethylene and acetic acid (Fig. 3(d)) also furnishes ethylene molecules for HCP mechanism (Fig. 3(a)) (Kozlowski and Davis 2013).

The experiment conducted over four hours of reaction, only H_2_ (85.7%) and CO (14.3%) were produced, accompanied by a substantial increase in the gas volume produced, reaching 3,325 mL. The peak of hydrogen formation under these conditions was reached at the five-hour reaction, with a content of 89.9% (3,039 mL). At this time, it is important to note that after H_2_, the second major gas was CH_4_ (4.5%), which began to increase, reaching 65.7% during the eight-hour experiment as the main gas component produced.

These changes in the gaseous fractions were possibly associated with alterations in catalyst chemistry. As previously discussed for the catalytic tests, where acetaldehyde decomposition to CH_4_ took place as the predominant reaction.

The gas volume continuously increased over time, reaching its maximum value at the eight-hour mark of the reaction, with 5,460 mL. The kinetic study was not extended beyond 8 h due to operational safety limitations, where the pressure could potentially exceed 80 bar, and there were concerns about the reactor material’s ability to withstand such conditions.

### Analysis and characterization of the liquid fraction

#### Determination of ethanol content

**Fig. 4:**
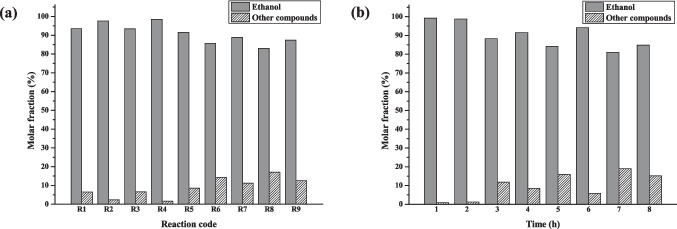
Ethanol and other compounds contents in liquid fractions after dehydrogenation reaction (**a**) Samples R1-R9 from conditions studies and (**b**) Samples from each experiment of the kinetic study at 280 °C with 10% of NiO

The evaluation of the liquid fractions’ contents resulting from the reactions was made using diverse techniques. Firstly, all samples were analyzed by GC-FID, and apart from residual ethanol, some compounds were observed. Before identifying the minor compounds, the remaining ethanol content in all fractions was determined by GC-FID, indicating the percentage of compounds as well formed. A calibration curve was constructed with different concentrations of ethanol in ethyl acetate. Considering 100% of ethanol at the beginning of the reaction, the ethanol percentage in each catalytic test (R1-R9) and kinetic experiment (1–8 reaction hours) samples were evaluated and presented at Fig. 4.

From these results, it was possible to establish some valuations, for instance, in the first set of experiments (R1, R2, and R3) at 240 °C, a slight reduction of ethanol content was observed, with an average of 5.15%. The second set of tests at 260 °C, the initial catalyst increment, from 6% (R4) to 10% (R5) resulted in a similar average consumption in ethanol content (5.08%), comparable to that observed in the first set (5.15%). However, the subsequent 4% catalyst increment led to a more substantial ethanol consumption, reaching 14.3% (R6). Comparing the reactions test with 14% of catalyst content, the 20 °C increase in reaction temperature had a considerable effect on ethanol consumption, rising from 6.6% (R3) to 14.3% (R6).

This significant increase in ethanol consumption is consistent with the gaseous phase results described, where a change in the distribution of produced gases was observed. From R3 to R6, CH_4_ became the major component, which originated from acetaldehyde decomposition (Eq. 8). This suggests that R6 conditions favored Eq. 8 reaction. It is also important to note that although H_2_ was not the major gas produced at R6, a considerable overall increase in gas volume was observed, aligning with ethanol dehydrogenation followed by acetaldehyde consumption.

The temperature increases to 280 °C did not result in a considerable change in ethanol consumption, with an average reduction of 13.6% for the last set of catalytic tests (R7, R8 and R9), which was similar to the one observed at R6 (14.3%). In general, it was observed that there was a tendency for ethanol consumption and among all conditions tested, R8 condition (at 280 °C with 10% of NiO) yielded 17.0% of ethanol consumption (Fig. 3(a)) as the best result.

Figure 3(b) represents the kinetic study results of ethanol consumption and production of the organic compounds, at 280 °C with 10% NiO catalyst from 1 to 8 reaction hours experiments. With two hours of reaction, a minor decrease in ethanol content was observed, from 0.82% (1 h experiment) to 1.28%.

Although the proposed intermediate molecules from Fig. 3(a) were not detectable in the liquid fraction, and ethylene remained absent in the gaseous fractions, a relative peak corresponding to ethyl acetate was observed in all liquid samples, despite being in low concentrations. This observation could indicate that the mechanism for propane synthesis during the initial reaction hours aligns with Fig. 3(b) and 3(c) proposals.

By the three-hour reaction experiment, a high reduction in ethanol content was observed, of 11.8%, which agreed with the composition results of the gaseous fraction, where H_2_ turned into the major gas produced, with a significant increase in volume (Fig. 2). A displacement to Eq. 4, which furnishes acetaldehyde also indicates a consequent decrease in ethanol content. A similar behavior was observed with a four-hour reaction of the kinetic study, while with five hours another considerable reduction in ethanol content was noted, to 15.8%, coinciding with the maximum peak of H_2_ production. Considering the experiments with 6, 7, and 8 h of reaction, an average ethanol consumption of 13.3% was observed.

#### Identification and quantification of minor components

**Fig. 5:**
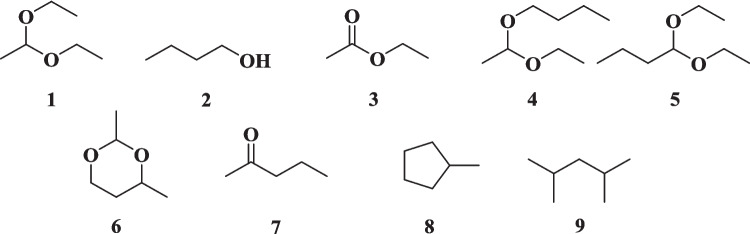
Minor components of liquid fraction suggested by GC–MS

To further identify the organic compounds formed in the liquid fractions during the reactions, a GC–MS analysis was conducted. Among the minor components, several oxygenated molecules were suggested, including 1,1-diethoxyethane (*m/z* 118) (**1**), 1-butanol (*m/z* 74) (**2**), ethyl acetate (*m/z* 88) (**3**), 1-(1-ethoxyethoxy)butane (*m/z* 118) (**4**), 1,1-diethoxybutane (*m/z* 146) (**5**), 2,4-dimethyl-1,3-dioxane (*m/z* 116) (**6**), 2-pentanone (*m/z* 86) (**7**). Additionally, hydrocarbons such as methylcyclopentane (*m/z* 84) (**8**) and 2,4-dimethylpentane (*m/z* 100) (**9**) were also suggested (Fig. 5).

The formation of the proposed compounds in the liquid fraction can be attributed to the presence of active acid/base sites on the catalyst used. These NiO catalyst sites facilitated the mechanisms of aldol condensation and nucleophilic addition, originating from acetaldehyde, which is one of the products of ethanol dehydrogenation reaction (Eq. 4) (Lopez-Olmos et al. 2020).

Among the organic compounds formed, three showed the highest intensities in the GC-FID analysis: 1,1-diethoxyethane (**1**), 1-butanol (**2**), and methylcyclopentane (**8**). Focusing on identification, in the first set of experiments (R1—R3), besides ethanol, it was observed mainly the presence of the compound suggested as 1,1-diethoxyethane (**1**). From the ^13^C NMR spectrum of R1 liquid sample, it was possible to observe the signals of ethanol at 56.8 and 17.4 ppm, and other signals at 99.4, 60.5, 19.2 and 14.5 ppm. These signals could be attributed to the methine, methylene, and methyl carbons, respectively, in accordance with expected signals for the suggested acetal **1** (Fig. S1 and S2 in Supplementary Information). The standard of **1** was acquired and by comparing GC-FID and NMR analyses, it was confirmed the presence of this compound in the reaction’s samples as one of the products from ethanol dehydrogenation.

A hydrocarbon was also observed in this set of experiments (R1-R3), two compounds, methylcyclopentane (**8**) or ethylcyclobutane, both with *m/z* 84, were suggested by GC–MS library. However, the content of this component was minimal, and consequently, the signals related to any of the suggested compounds were not found in the spectroscopic analyses. So, it was decided to acquire the compound with the largest similarity, which was methylcyclopentane (**8**). After GC-FID analysis, it was possible to confirm, that the compound present in the sample was not **8**, indicating that it could potentially be one of its isomers, such as ethylcyclobutane.

Another compound that stands out in intensity in the samples was 1-butanol (**2**), as suggested by the GC–MS library, which was detected from R6 to R9. From R8 ^13^C NMR spectrum, in addition to the signals of acetal **1** and ethanol, it was possible to observe signals at 62.1, 34.9, 19.1 and 13.6 ppm. Compared with 1-butanol standard ^13^C NMR spectrum, it was possible to confirm this compound at the mentioned samples (Fig. S3 in Supplementary Information). 1-Butanol standard GC-FID analysis yielded an identical retention time as the compound observed in the samples, further supporting its identification by coelution analysis.

**Fig. 6:**
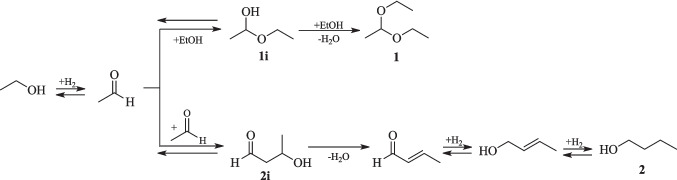
Proposed mechanism for synthesis of 1,1-diethoxyethane (**1**) and 1-butanol (**2**)

The proposed mechanism for the 1,1-dietoxyethane (**1**) formation involves the following steps: a) ethanol dehydrogenation to acetaldehyde; b) nucleophilic addition of ethanol to acetaldehyde, resulting in the hemiacetal 1-ethoxyethanol (**1i**); c) acid-catalyzed water elimination and a second nucleophilic addition of ethanol, furnishing **1**. The 1-Butanol (**2**) started to be formed only at R6 condition, and according to the literature, the formation of **2** from ethanol in this catalytic process is known as Guerbet reaction (Kozlowski and Davis 2013). This mechanism involves a) ethanol dehydrogenation to acetaldehyde; b) aldol condensation to acetaldol (**2i**); c) dehydration of acetaldol to 2-butenal; d) hydrogenation to butyraldehyde; and e) hydrogenation to 1-butanol (**2**) (Fig. 6) (Inui et al. 2004; Kozlowski and Davis 2013; Lopez-Olmos et al. 2020).

The 1-butanol (**2**) synthesis mechanism from ethanol is widely observed when MgO is used as a metal oxide, due to its basic character, where aldol condensation occurs (Kozlowski and Davis 2013; Lopez-Olmos et al. 2020; Ndou et al. 2003). Therefore, the observation of 1-butanol (**2**) from the use of NiO as a catalyst agrees with the study of Lopez-Olmos et al. (2020). Interestingly, the observation of acetal **1** indicates that the synthesized NiO catalyst also has acid sites, which are responsible for promoting the acetaldehyde acetylation reaction with ethanol, furnishing the hemiacetal 1-ethoxyethanol (**1i**) (Inui et al. 2004; Lopez-Olmos et al. 2020; Pang et al. 2016). The high ethanol content in the reaction facilitates nucleophilic addition in the following steps, contributing to the formation of **1**.

The concentration of the identified compounds—1,1-diethoxyethane (**1**), and 1-butanol (**2**)—were determined in the catalytic tests (R1-R9) and kinetic study (1–8 h-reaction experiments) samples. By employing external calibration curves with standards of **1** and **2,** the content (%) of these compounds, as well as that other compounds were calculated and are shown in Table 3 and 4.

**Table 3:** Ethanol, 1,1-diethoxyethane (**1**), 1-butanol (**2**) and other compounds contents in liquid fractions after dehydrogenation reaction from the catalytic tests’ samples R1-R9

Reactions	Ethanol (%)^a^	Ethanol Consumption (%)^b^	Compounds content (%)		
			**1**	**2**	**Others**^**c**^
R1	93.52	6.48	1.80	0.00	4.67
R2	97.61	2.39	0.06	0.00	2.33
R3	93.41	6.59	2.29	0.00	4.29
R4	98.37	1.63	0.15	0.00	1.48
R5	91.48	8.52	0.23	0.00	8.28
R6	85.71	14.29	3.16	1.12	10.01
R7	88.80	11.20	2.40	0.67	8.13
R8	82.96	17.04	2.68	1.70	12.65
R9	87.40	12.60	2.30	0.95	9.35

^a^ Content of residual ethanol determined from liquid fractions;

^b^ Content of consumed ethanol by conversion into other compounds trough the reaction;

^c^ Other compounds content calculated from deduction of compounds **1** and** 2** contents from ethanol consumption content

**Table 4:** Ethanol; 1,1-diethoxyethane (**1**); 1-butanol (**2**), and other compounds contents in liquid fractions after dehydrogenation reaction from the kinetic study reactions (280 °C, 10% NiO)

Reaction time (h)	Ethanol (%)^a^	Ethanol Consumption (%)^b^	Compounds content (%)		
			**1**	**2**	**Others**^**c**^
1	99.18	0.82	0.04	0.01	0.77
2	98.72	1.28	0.05	0.01	1.23
3	88.21	11.79	2.87	0.11	8.80
4	91.48	8.52	0.23	0.01	8.27
5	84.19	15.81	4.69	0.89	10.23
6	94.17	5.83	1.20	0.12	4.52
7	81.04	18.96	3.15	1.32	14.49
8	84.89	15.11	3.21	0.82	11.09

^a^ Content of residual ethanol determined from liquid fractions;

^b^ Content of consumed ethanol by conversion into other compounds trough the reaction;

^c^ Other compounds content calculated from deduction of compounds **1** and** 2** contents from ethanol consumption content

According to Table 3, the average content of 1,1-diethoxyethane (**1**) was 1.38%, for the set of experiments R1-R3. The increment in temperature, from 240 °C to 260 °C (R4-R6) was not significant compared to the average of the first set (R1-R3). However, comparing R3 to R6, there was observed an increase of 0.87% for compound **1**. This behavior is in accordance with the gaseous phase data, since even the proportion of hydrogen in relation to the other gases produced has decreased in comparison with R3 conditions, at R6 a significant increase in the volume of hydrogen production was observed. Consequently, in the liquid fraction, there was an increase in the consumption of ethanol with a higher concentration of acetaldehyde, which contributed to the formation of **1**. The higher concentration of acetaldehyde in liquid fraction favored the production of CO and CH_4_ throughout the decarbonization mechanism (Eq. 8), within each route, resulting in 14.29% of ethanol consumption at R6.

1-Butanol (**2**) was not observed from R1 to R5, only at R6 to R9 tests. Besides the change in the major gas produced at R6 test the increase of hydrogen volume and temperature favored the production of 1.12% of 1-butanol. Since more hydrogenation and water elimination steps are required for Guerbet reaction (Fig. 6), generally, high temperatures are needed to achieve the conversion of ethanol to 1-butanol with good yields and selectivity. From the Guerbet process, 1-hexanol and 1-octanol also can be formed with following condensation steps with ethanol molecules, but in our study, these compounds were not detected. Although it is known that bifunctional catalysts have better performance for Guerbet reaction, especially those which have copper in its composition, the NiO catalyst developed in this study proved to be useful for this reaction conversion (Lopez-Olmos et al. 2020).

With the appearance of 1-butanol in R6 test, a Karl Fischer analysis was carried out to determine if there was a difference in the water content when compared to the R3 test, due to the dehydration step necessary to produce **2**, as described at Fig. 6. So, considering the water content of ethanol used for the reaction as zero at the beginning, at R3, 1.69% was detected and at R6, 9.38%, proving a water increment.

For the last set of reactions (R7-R9) at 280 °C (Table 3), 2.46% average of acetal **1** and 1.11% average of 1-butanol (**2**) formation was observed. These results were similar to those obtained under R6 condition (3.16% of **1**, 1.12% of **2**, and 14.29% of ethanol content conversion). It can be noticed that the variation of catalyst content did not result in an increase of either acetal **1** or 1-butanol **2** formation at 280 °C. However, a continuous increase in H_2_ volume, as observed from R6 indicates an increase in ethanol dehydrogenation, consequently resulting in more acetaldehyde molecules to produce **1**, and for aldol condensation leading to 1-butanol (**2**). These compounds, subsequently contributed to the generation of CO and CH_4_ in the gas phase.

Analyzing the kinetic study’s liquid samples (Table 4), at the two-hours reaction experiment due to the small ethanol consumption content and hydrogen production, the formation of acetal **1** and 1-butanol (**2**) was not prominent. From the 3rd hour on, a higher content and volume of hydrogen was produced, along with 11.79% ethanol reduction content in the liquid fraction, where 1,1-dietoxyethane (**1**) represents 2.87%, but the butanol (**2**) content was still very low (0.11%). The peak of hydrogen production was observed at the five-hour experiment, with a significant consumption of ethanol content of 15.81%. This time also marked the highest content of acetal **1**, with 4.69%, while 1-butanol (**2**) content was 0.89%. Contrary to the production of acetal **1**, which begins with few hours of reaction, the formation of 1-butanol (**2**) appeared to depend on extended reaction times, since more reaction steps are necessary for its formation. The peak of 1-butanol production was observed at a seven-hour reaction, reaching 1.32%. Once again, the Karl Fischer analysis of liquid samples from three and seven-hour experiments indicated an increase in water content, from 8.50% to 11.02%, which agrees with 1-butanol formation.

Comparing the results of seven-hour and eight-hour reactions, although there was an increase in the volume of gas produced, the major component changed from H_2_ to CH_4_. As discussed earlier, CH_4_ is generated from acetaldehyde decarbonization (Eq. 8), indicating that time is an important feature for this reaction. Although 15.11% of ethanol conversion content was observed at the eight-hour reaction, it was lower when compared to the seven-hour reaction (18.96%), which might indicate that the conversion rate of ethanol content was less significant when CH_4_ is the major component than when hydrogen is the major gas of the gaseous fraction. Interestingly, the influence of time did not impact the formation of acetal (**1**) and 1-butanol (**2**) in same proportion, which presented levels like those observed in the seven-hour reaction.

**Fig. 7:**
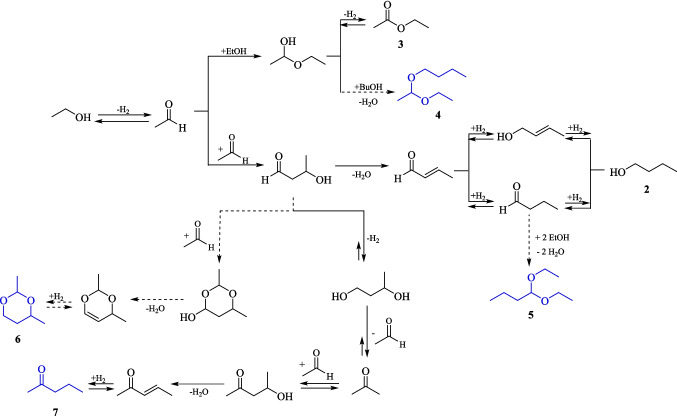
Proposed mechanism for synthesis of 1-(1-ethoxyethoxy)butane (**4**), 1,1-diethoxybutane (**5**), 2,4-dimethyl-1,3-dioxane (**6**), and 2-pentanone (**7**)

As previously mentioned, among the organic compounds identified in the liquid phase, as presented in Fig. 5, are suggested: ethyl acetate (**3**), 1-(1-ethoxyethoxy)butane (**4**), 1,1-diethoxybutane (**5**), 2,4-dimethyl-1,3-dioxane (**6**), 2-pentanone (**7**), and two hydrocarbons **8** and **9**. In addition to acetal **1** and 1-butanol (**2**), the presence of ethyl acetate (**3**), 2-pentanone (**7**), and 2,4-dimethylpentane (**9**) was confirmed using commercial standards for GC-FID analysis. While the other compounds were not verified using standards, their formation is feasible under the reaction conditions according to the proposed mechanisms presented at Fig. 7.

In addition to the compounds mentioned by GC-FID and GC–MS analysis, at R1 and R8 ^13^C NMR spectra, additional signals were detected indicating the presence of another compound, not detected by the gas chromatography analyses. This compound has approximately one-third of the relative proportion of the identified acetal **1**. In the ^13^C/DEPT NMR spectrum, the signals at 93.8, 60.5, and 22.6 ppm correspond to a methine, methylene, and methyl carbon, respectively, similar to the signals observed for acetal **1** (Fig. S4 in Supplementary Information). Thus, the unknown compound possibly has a similar structure of acetal **1**, but it could not be one of the other acetals 1-(1-ethoxyethoxy)butane (**4**) or 1,1-diethoxybutane (**5**). Compounds **4** and **5** eluted after **1**, in a very low intensity compared to **1**, which did not corroborate with the intensity of the signals at ^13^C NMR spectrum. Based on these assumptions, a hypothesis emerged that the unknown compound might have coeluted with ethanol, which is why it was not detected by gas chromatographic analyses. Therefore, considering its molar mass is lower than that of acetal **1**, it was suggested that the compound could be the hemiacetal 1-ethoxyethanol (**1i**)—intermediate formed by the aldol addition previously at the dehydration step (Fig. 6). This suggestion is supported by R1 and R8 ^1^H NMR spectra, where despite the high concentration of ethanol, it was possible to observe some signals related to acetal **1** in R1 ^1^H NMR spectrum, which was compared to the ^1^H NMR of the standard, as the presence of a quartet at 4.69 ppm (^3^* J* = 5.3 Hz) attributed to CH, one of the multiplets from 3.45 to 3.54 ppm related to one of the CH_2_, and a duplet at 1.28 ppm (^3^* J* = 5.3 Hz) for the CH_3_ group, attached to the CH (Fig. S5 in Supplementary Information). For the suggested compound 1-ethoxyethanol (**1i**), there is a large quintet at 4.81 ppm (^3^* J* = 6.0 Hz), explained by the splitting of CH with -CH_3_ and -OH hydrogens nuclear spins, added a multiplet from 3.75 to 3.82 ppm, consistent to carbinolic CH_2_ group, a triplet at 1.35 ppm (^3^* J* = 7.0 Hz) for CH_3_ linked to the CH_2_, and a duplet at 6.45 ppm (^3^* J* = 7.3 Hz) for the OH coupling with CH (Fig. S6 in Supplementary Information). So, it was suggested that a significant part of remaining content defined as a mixture of all other compounds in Fig. 4, and Tables 3 and 4 are represented by the hemiacetal 1-ethoxyethanol (**1i**).

### Analysis and characterization of the used catalyst from kinetic study

**Fig. 8:**
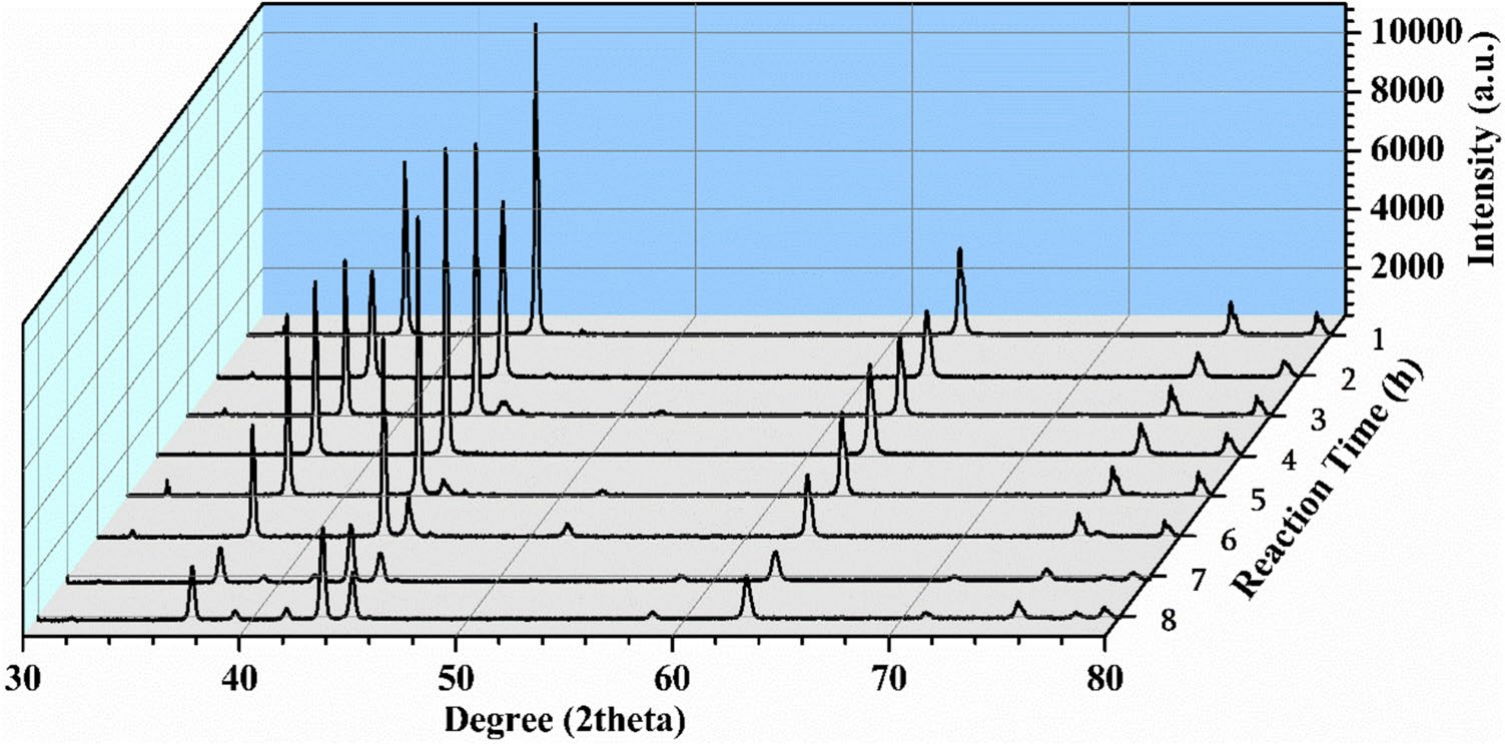
XRD analysis of the NiO catalyst recovered after the reactions of the kinetic studies from one hour to eight-hour experiments

Figure 8 presents the XRD analysis of the catalyst recovered after each experiment of kinetic study, from one hour to eight hours. This analysis aims to verify the changes in the crystalline structure of the catalyst after the performed reaction.

The diffraction peaks corresponding to the face-centered cubic (FCC) structure of NiO were observed in the recovered catalyst from all kinetic experiments. The NiO FCC characteristic peaks at 37° (111), 43° (200), 63° (220), 75° (311), and 79° (222) (JCPDS 004–0850) were observed, which suggested the preservation of the synthesized crystalline structure of the catalyst, for 8 h (Bach et al. 2022).

A substantial decrease in the intensity of NiO FCC peaks was observed at XRD analysis of the five-hours reaction experiment (Fig. 8). This is observed due to the formation of two new crystalline phases related to Ni metallic. Starting at seven-hours reaction, peaks at 39.3° (010), 41.6° (002), 44.6° (200), 59.6° (012), and 71.1° (110) were observed, related to the hexagonal close-packed (HCP) structure of Ni^0^ (JCP-045–1027). The second group of peaks at 51.8° (200), 78.2° (222) represents the characteristic peaks of the face-centered cubic (FCC) structure of Ni^0^ (JCP-004–0850) (Bach et al. 2022).

All characteristic peaks of Ni^0^ HCP structure appeared at the seven-hour reaction experiment. However, at the three-hour reaction, one of the mentioned peaks (44.6°) was already detected, as well as the peak at 51.8^o^_,_ which corresponds to the Ni^0^ FCC structure, but in low intensity. These observations indicated that the reduction of Ni^2+^ from NiO FCC structure by H_2_ atmosphere was already occurring, resulting in Ni^0^ FCC, which subsequently transitioned to Ni^0^ HCP structure.

As previously discussed, in the gaseous phase, during the three-hour reaction experiment, H_2_ was the preferred gas formed, with a significant increase in volume. In the liquid sample, at this time, the first significant ethanol conversion content with the formation of acetal **1** was also observed. So, according to the XRD analysis, it can be suggested that the first shift from NiO FCC to Ni^0^ FCC favored the mentioned reactions.

From these data, the fresh NiO FCC nanoparticles might have primarily contributed to the propane production. As the Ni^0^ FCC structure became more pronounced, H_2_ turned into the predominant gas in the gaseous phase, indicating a preference for hydrogen production. At a six-hour reaction, the peak at 51.8° was observed, where during this period, H_2_ remained the major gas in the gaseous phase. Following this, at the next test with seven hours, a decline in H_2_ content was observed, while CH_4_ content increased. However, the total gas volume continued to grow. This significant increase in the CH_4_ content was likely favored by the transition from Ni^0^ FCC to Ni^0^ HCP structure from decarbonization of acetaldehyde, where at seven-hour reaction, the peak referent to Ni^0^ FCC (51.8°) disappeared and the other peaks of the Ni^0^ HCP (39.3°, 41.6°, 59.6°, and 71.1°) were fully detected by XRD analysis.

This transition behavior of metallic nickel crystalline phases from FCC to HCP was also discussed in the previous study Bach et al. (2022). XRD analysis indicated these changes, which were confirmed by high-resolution transmission electron microscopy (HRTEM) images. It was observed that the change from Ni^0^ FCC, initially present within the three-hour reaction, progressed to a Ni^0^ HCP arrangement by the five-hour reaction (Bach et al. 2022).

Nickel particles with different sizes and crystalline structures can be prepared using various methods, such as chemical reduction, sputtering, thermal procedures, reverse microemulsion technique, and polyol-mediated processes (Sevonkaev et al. 2014). The transition phenomenon between FCC and HCP crystalline structure phases of metallic nickel, observed in the ethanol dehydrogenation kinetic study, can be attributed to several aspects. These aspects include the selected temperature range (280 °C), reaction time, and formation of linear acetal compounds in the liquid phase: 1,1-diethoxyethane (**1**), 1-(1-ethoxyethoxy)butane (**4**), and 1,1-diethoxybutane (**5**).

Sevonkaev et al. (2014) investigated the conversion of NiO from FCC to a HCP structure, using a variety of polyol compounds. XRD analysis of nickel particles was utilized to determine the percentage of conversion to the HCP phase. At a temperature of 275 °C for a duration of 18 h, the diols, propanediol (PD), and ethylene glycol (EG), exhibited minimal conversion from NiO FCC to Ni^0^ HCP phase. Specifically, the conversion was negligible for PD and ranged from 7 to 10% for EG. In this context, the hydroxyl group of EG was responsible only for the reduction of Ni^2+^ to Ni^0^, without inducing substantial crystalline transformation. Instead, the Ni^0^ FCC crystalline structure predominantly crystallized in an HCP structure only on the catalyst’s surface. The same test was done with higher boiling point glycols, like di- tri- and tetra-ethylene glycol, and a significant conversion rate of 95% to the HCP structure was detected. It was observed that a high conversion rate of Ni crystal phases takes place when employing linear poly-glycol containing an ether fragment (–O–) (Sevonkaev et al. 2014).

**Fig. 9:**
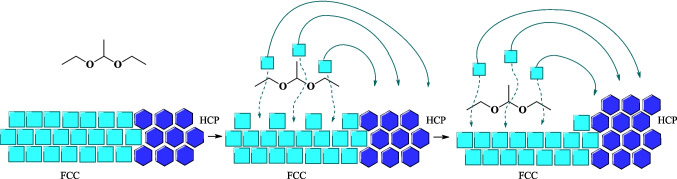
Proposed mechanism of the rearrangement of Ni atoms from an FCC to an HCP crystal structure promoted by 1,1-dietoxyethane based on Sevonkaev et al. (2014) study

Based on these results, a mechanism was proposed by the authors, where the ether fragment is responsible for dislocating and transporting Ni atoms from FCC location to a new location, where a HCP structure rearrangement occurred (Sevonkaev et al. 2014). In our study, this transition was promoted by 1,1-dietoxyethane, as shown on Fig. 9. A surface diffusion or dissolution-reprecipitation could be involved in this process. Furthermore, an increase in the number of ether fragments indicated a higher intensity of HCP structure peaks in the XRD analysis, which suggests enhanced efficiency in the transition mechanism.

Further correlations can be established between the results of the gas and liquid compositions with the XRD analysis. At the three-hour reaction, hydrogen became predominant, with a more substantial gas volume when compared to the two-hour reaction. Consequently, a peak from FCC structure of Ni metallic began to appear in the XRD analysis, indicating the reduction of NiO FCC by H_2_ atmosphere. Moreover, a higher concentration of H_2_ also suggested a tendency toward acetaldehyde production and consequent consumption of ethanol content in the liquid phase, with 11.79% of ethanol conversion. Therefore, this ethanol turned into acetaldehyde followed by the mechanisms described in Fig. 3, Fig. 6 and 7, other organic compounds were observed, such as 2.87% of acetal **1**.

The synthesized acetal **1** has two ether fragments, which by the Sevonkaev et al. (2014) proposed mechanism, it facilitated the conversion of the developing metallic nickel FCC structure into HCP crystal phase. It is worth noting that not only H_2_ can promote the reduction of NiO, but also ethanol participates in this process due to the presence of its hydroxyl group. From this point onward, maintenance of this behavior was observed. In other words, high H_2_ content led to the NiO FCC structure reduction to Ni^0^ FCC intermediate structure, and with acetaldehyde presence in the liquid phase, there is a continuous conversion of ethanol content to acetals **1**, **4** and **5**, which facilitated the transition of Ni^0^ FCC intermediate structure to HCP crystalline phase. This transition continued until all Ni^0^ FCC intermediate structure was completely converted into HCP crystalline structure, over the course of seven-hour reaction.

Then, an increase in CH_4_ content was observed, which derived from decarbonization of acetaldehyde (Eq. 8). This process also led to a conversion of ethanol content to acetal **1**, 1-butanol (**2**) among other organic compounds.

## Conclusions

In summary, the new exploratory study employing the green-synthesized NiO nanoparticles catalyst for ethanol dehydrogenation yielded interesting results in relation to the three assessed phases: gaseous, liquid, and solid fractions. A new set of reaction conditions was evaluated with three levels of catalyst concentrations (6%, 10%, and 14% NiO) and temperatures (240, 260 and 280 °C). The highest selectivity for hydrogen (97.4%) in the gaseous phase was observed at condition R5 (10% NiO at 260 °C), but the tests conducted at 280 °C resulted in a substantial volume of gases produced, reaching 3,325 mL at condition R8 (10% NiO at 280 °C). While the hydrogen percentage decreased, the overall gas volume remained considerably high, at 710 mL. In general, the influence of temperature increase was also observed on the liquid samples, with a significant ethanol conversion content achieved. Among other organic compounds, the 1,1-dietoxyethane and 1-butanol were preferentially formed.

With the calculated calorific power, R8 reaction condition was selected for evaluating the reaction over time. Interestingly, a transition in the major component of the gaseous phase was observed. At the beginning, propane was preferentially formed, with a transition to hydrogen and then to methane. The DRX analysis of the catalyst recovered after the kinetic reaction tests provided insights into the mechanism, where changes on the crystal structure that aligned with the observed gas and liquid phase changes.

This study underscores the potential of a green-synthesized NiO catalyst for efficient ethanol dehydrogenation, offering insights into the dynamic nature of the reaction and its implications for hydrogen production, and other fuel gases, the synthesis of valuable organic compounds, as well as the promotion of metallic nickel with a hexagonal close-packed (HCP) crystal phase. The observed compounds on the liquid phase, as 1,1-dietoxyethane and 1-butanol indicate the catalyst’s efficiency in aldol condensation mechanisms involving acid/basic sites. Principally the formation of the acetal 1,1-dietoxyethane, which has 2 ethers fragments, possibly involved in the crystalline structure changing mechanism.

Further studies can extend these findings by applying ethanol as a substrate to calculate carbon conversion content into the observed organic molecules in the liquid phase. Furthermore, exploring other substrates, such as ethylene glycol, with the same NiO nanoparticles catalyst for the dehydrogenation reaction, will potentially produce hydrogen and other fuel gases in the gaseous fraction and other interesting and valuable organic compounds in the liquid phase.

## Supplementary Information

Below is the link to the electronic supplementary material.

Supplementary file1 (DOCX 1.24 KB)

## Data Availability

The authors declare that the data supporting the findings of this study are available within the paper and its Supplementary Material. Should any raw data files be needed in another format they are available from the corresponding author upon reasonable request.
