# Supplementary material for: Renewable fuel gases and important organic compounds production from ethanol dehydrogenation using nickel oxide, a green-synthesized catalyst
Source: Environ Sci Pollut Res Int. 2026 Apr 16;33(14):6861–76. doi: 10.1007/s11356-026-37421-8 (PMC13124856; doi:10.1007/s11356-026-37421-8)

**Supporting Information**

**Renewable fuel gases and important organic compounds production from ethanol dehydrogenation using nickel oxide, a green-synthesized catalyst**

Carla Maria Beraldi Gomes^a^, Denise Aparecida Zempulski^a^, Caroline da Ros Montes D’Oca^b^, Helton José Alves^a^

^a^ Renewable Materials and Energy Laboratory (LABMATER), Federal University of Parana (UFPR - Setor Palotina), R. Pioneiro, 2153, Dallas, Palotina, PR, Brazil.

^b^ Medicinal and Agrochemical Organic Synthesis Research Group (SOMA), Federal University of Parana (UFPR), R. Col. Francisco H. dos Santos, 100, Jardim das Américas, Curitiba, PR, Brazil

**Table S1.** Calculated calorific power (kJ) from R1-R9 ethanol dehydrogenation tests.

| Reaction code | Molar Fraction (%) | | | | Total gas volume (L) | Reactor pressure (atm) | Produced mass (g)* | | Calorific power (kJ) | | |
| --- | --- | --- | --- | --- | --- | --- | --- | --- | --- | --- | --- |
|  | **H_2_** | **CO_2_** | **CO** | **CH_4_** |  |  | **H_2_** | **CH_4_** | **H_2_** | **CH_4_** | **Total** |
| R1 | 70.1 | 0 | 0 | 29.9 | 292.3 | 2.0 | 0.034 | 0.114 | 4.8 | 6.3 | 11.1 |
| R2 | 85.3 | 14.7 | 0 | 0 | 328.9 | 2.0 | 0.046 | 0 | 6.5 | 0 | 6.5 |
| R3 | 82.5 | 1.5 | 2.8 | 13.2 | 310.6 | 3.9 | 0.082 | 0.105 | 11.6 | 5.8 | 17.4 |
| R4 | 85.7 | 0 | 14.3 | 0 | 475.0 | 2.0 | 0.067 | 0 | 9.5 | 0 | 9.5 |
| R5 | 97.4 | 0.6 | 2.0 | 0 | 835.0 | 3.9 | 0.261 | 0 | 37.0 | 0 | 37.0 |
| R6 | 22.2 | 0 | 14.2 | 63.4 | 1,385.0 | 2.0 | 0.051 | 1.153 | 7.2 | 64.0 | 71.2 |
| R7 | 18.8 | 0 | 50.0 | 31.2 | 3,210.0 | 12.8 | 0.645 | 8.615 | 91.4 | 478.1 | 569.5 |
| R8 | 21.3 | 2.5 | 20.3 | 55.8 | 3,325.3 | 15.8 | 0.935 | 19.803 | 132.5 | 1,099.1 | 1,231.6 |
| R9 | 16.5 | 3.0 | 19.2 | 61.3 | 3,332.6 | 15.8 | 0.718 | 21.814 | 102.9 | 1,210.7 | 1,313.6 |

*Calculated using Eq. 1, with *R*=0.082 atm L mol^-1^ K^-1^; *T*=298K; for H_2_: *a=* 0.242 atm L^2^ mol^-2^ and *b*=0.02651 L mol^-1^; for CH_4_: *a=* 2.27 atm L^2^ mol^-2^ and *b=*0,04301 L mol^-1^.


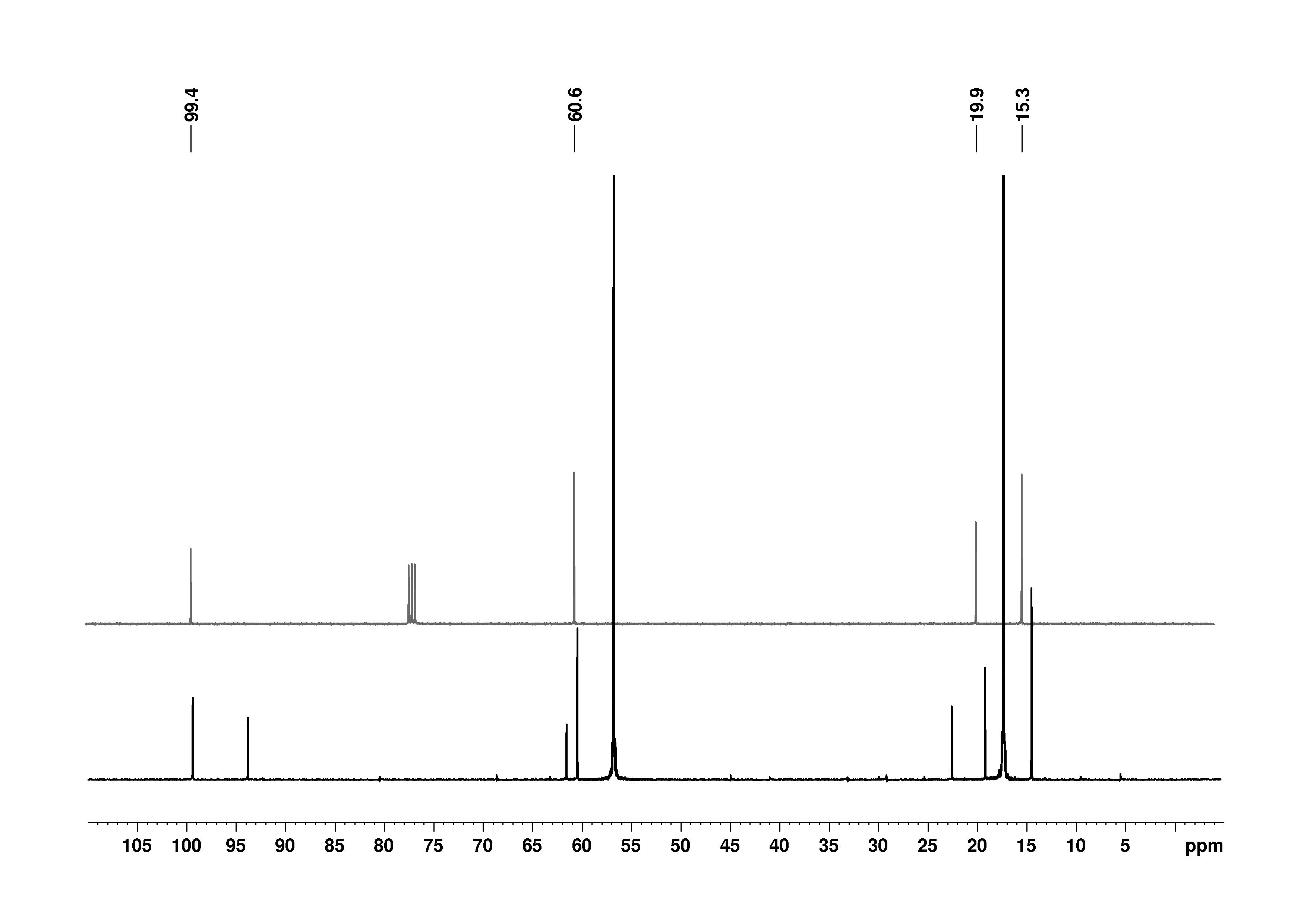


**(a)**

**(b)**

**Fig. S2** **(a)** ^13^C{H} NMR (150 MHz, CDCl_3_) of 1,1-dietoxyethane standard **(b)**^13^C{H} NMR (150 MHz) of R1 liquid fraction.

**Fig. S1** ^13^C{H} NMR (150 MHz) of R1 liquid fraction.


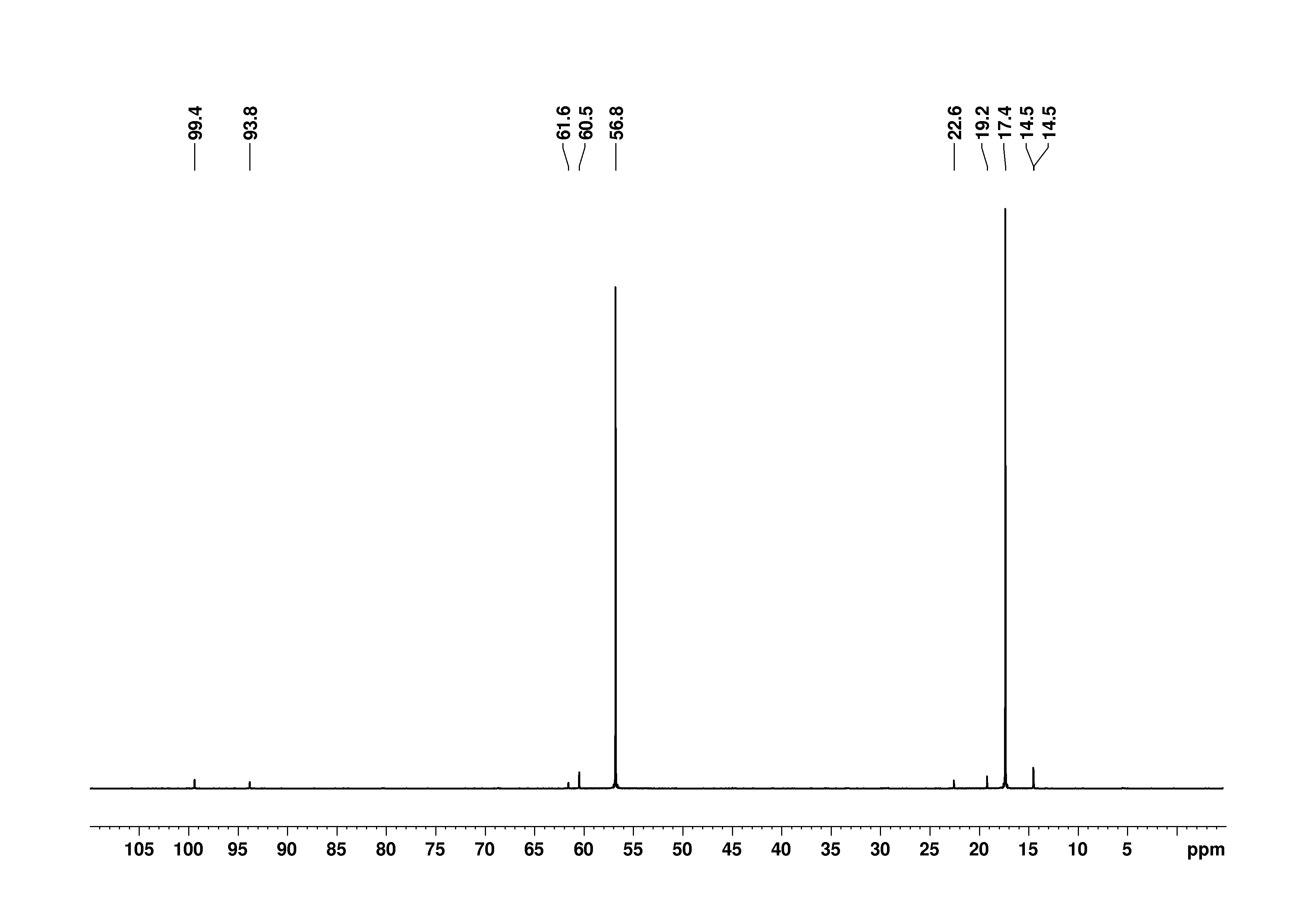

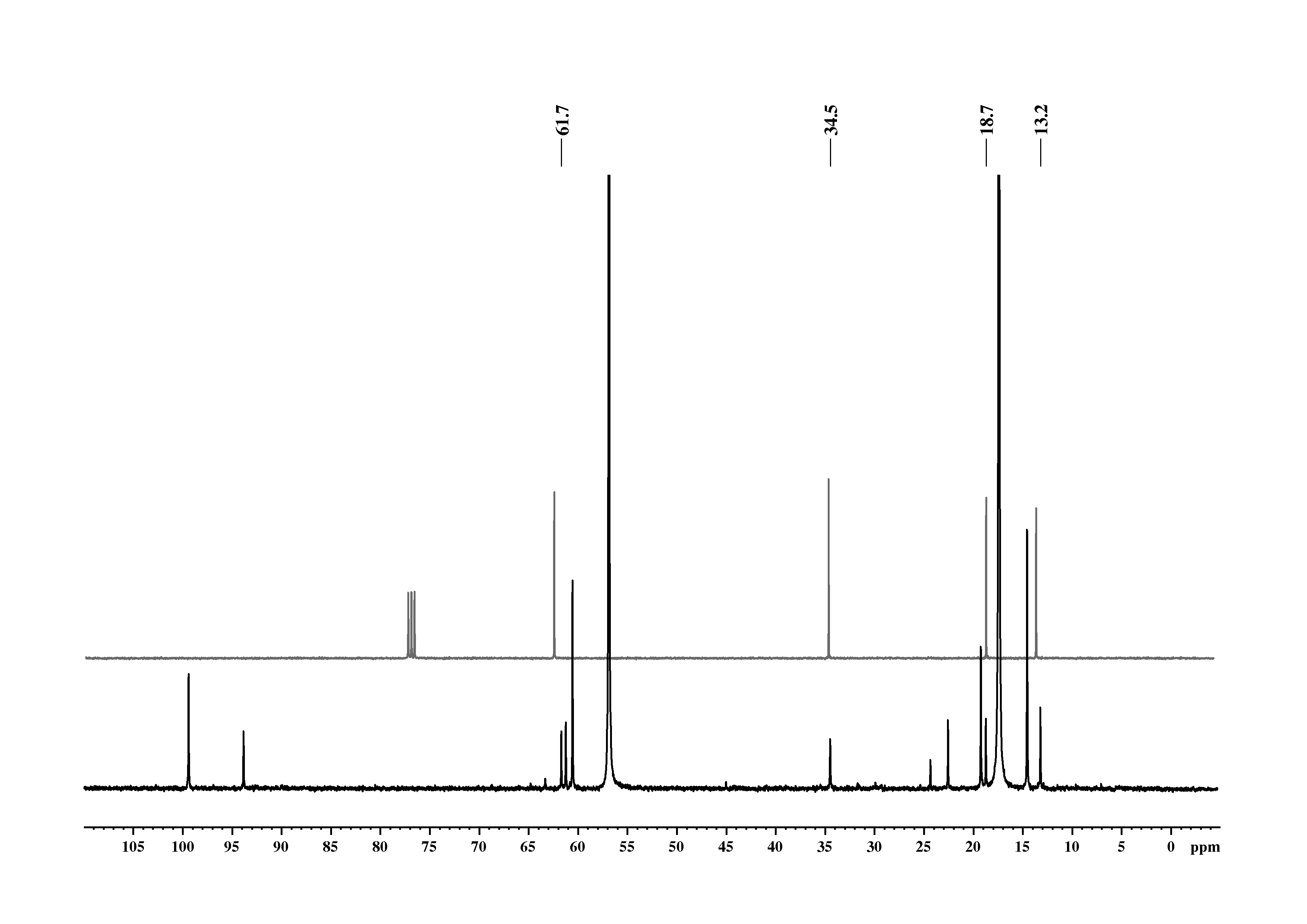


**(a)**

**(b)**

**Fig. S3** **(a)** ^13^C{H} NMR (150 MHz, CDCl_3_) of 1-butanol standard **(b)**^13^C{H} NMR (150 MHz) of R8 liquid fraction.


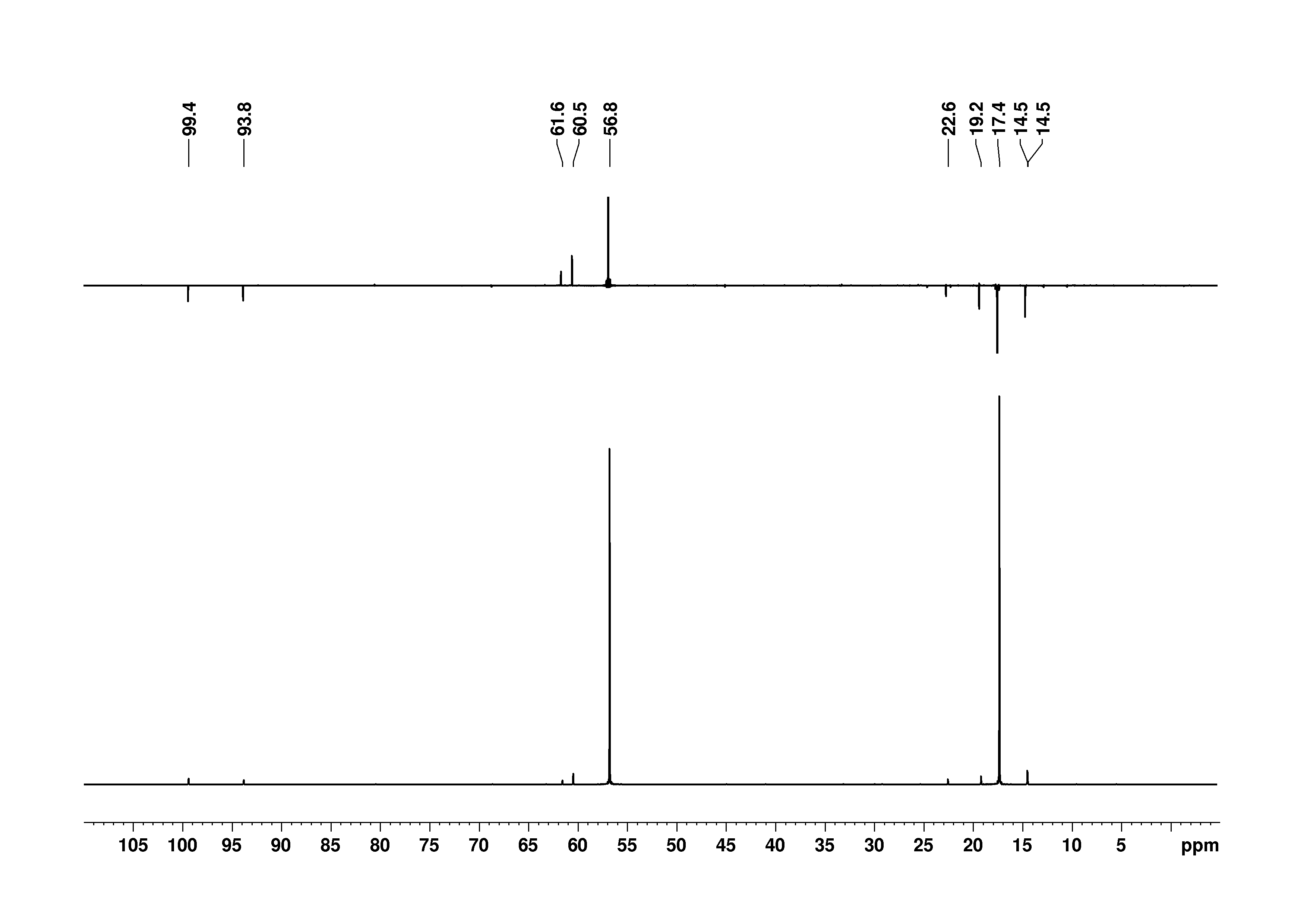


**Fig. S4** ^13^C{H}/DEPT NMR (150 MHz, CDCl_3_) of R1 liquid fraction.


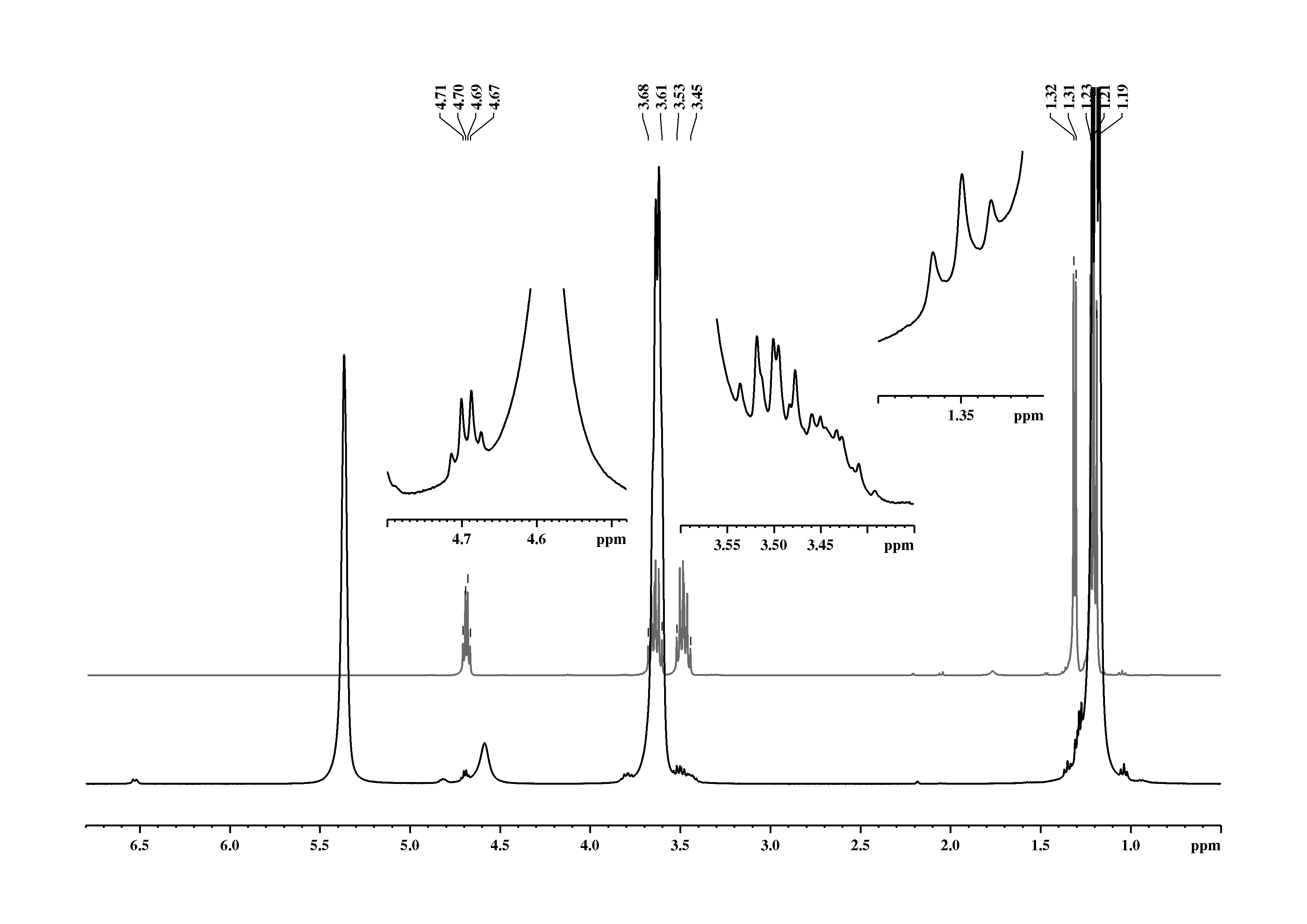


**(a)**

**(b)**

**Fig. S5** **(a)** ^1^H NMR (400 MHz, CDCl_3_) of 1,1-dietoxyethane standard **(b)**^1^H NMR (400 MHz) of R1 liquid fraction with expansions.

**Fig. S6** ^1^H NMR (400 MHz) of R1 liquid fraction with expansions.


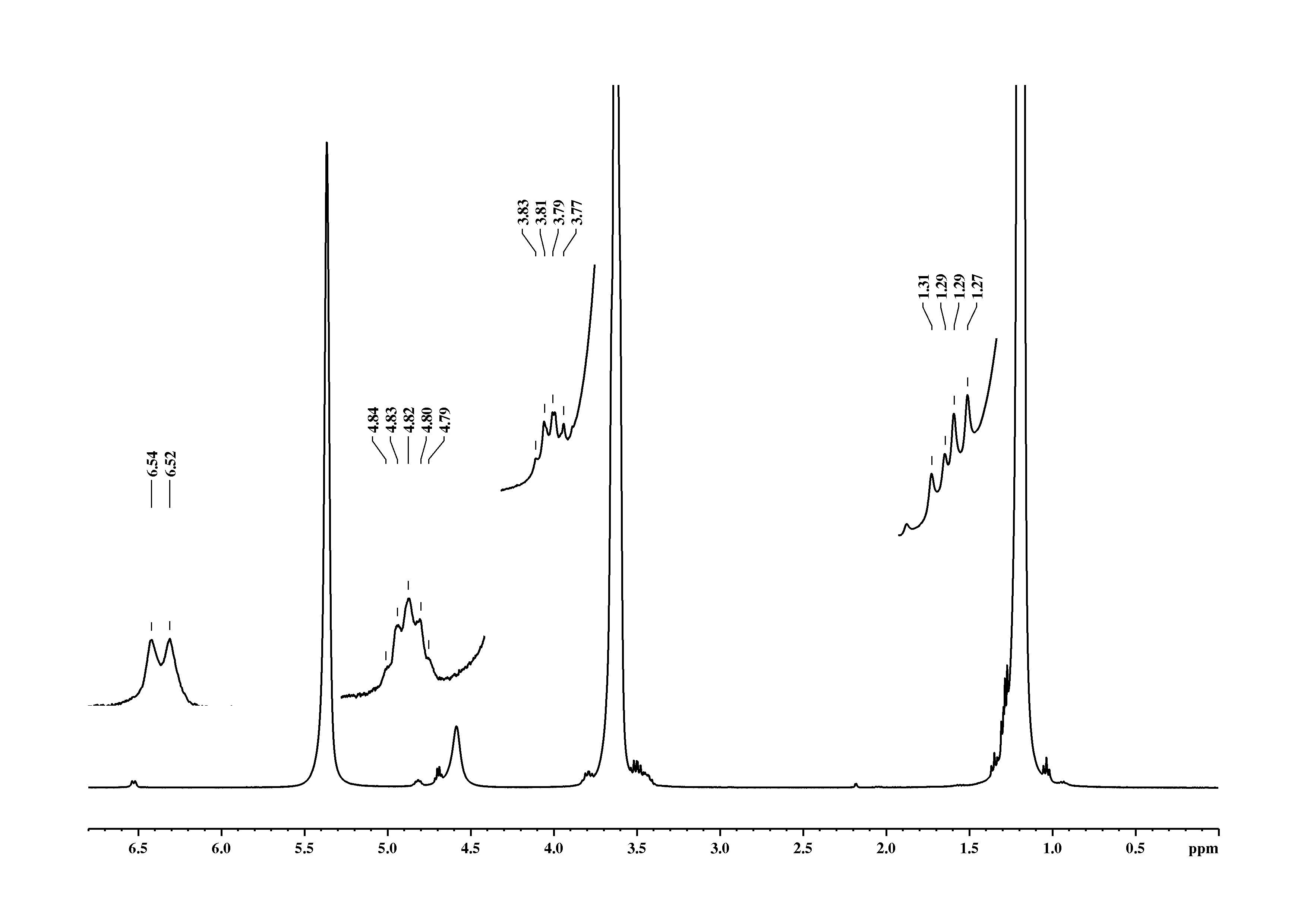

Supplement: Supplementary file 1 — Supplementary file1 (DOCX 1.24 KB) [file 11356_2026_37421_MOESM1_ESM.docx]
